# The Polymorphism rs17300539 in the Adiponectin Promoter Gene Is Related to Metabolic Syndrome, Insulin Resistance, and Adiponectin Levels in Caucasian Patients with Obesity

**DOI:** 10.3390/nu15245028

**Published:** 2023-12-07

**Authors:** Daniel de Luis Roman, Olatz Izaola Jauregui, David Primo Martin

**Affiliations:** Endocrinology and Nutrition Research Center, School of Medicine, Department of Endocrinology and Nutrition, Hospital Clinico Universitario, University of Valladolid, 47130 Valladolid, Spain; oizaola@saludcastillayleon.es (O.I.J.); dprimoma@saludcastillayleon.es (D.P.M.)

**Keywords:** *ADIPOQ* gene, obesity, metabolic syndrome, rs17300539

## Abstract

**Background and Aims**: The present study was designed to investigate SNP rs17300539 in the *ADIPOQ* gene and its relationships with obesity, metabolic syndrome (MS), and serum circulating adiponectin. **Methods**: The present design involved a Caucasian population of 329 subjects with obesity. Anthropometric and adiposity parameters, blood pressure, biochemical parameters, and the percentage of patients with metabolic syndrome were recorded. The *ADIPOQ* gene variant (rs17300539) genotype was evaluated. **Results**: The percentage of patients with different genotypes of the rs17300539 polymorphism in this sample was 86.0% (n = 283) (GG), 11.2% (n = 37) (GA), and 2.7% (n = 9) (AA). The allele frequency was G (0.76) and A (0.24). Applying the dominant genetic model (GG vs. GA *+* AA), we reported differences between genotype GG and genotype GA *+* AA for serum adiponectin levels (Delta: 7.5 ± 1.4 ng/mL; *p* = 0.03), triglycerides (Delta: 41.1 ± 3.4 mg/dL; *p* = 0.01), fastingcirculating insulin (Delta: 4.9 ± 1.1 mUI/L; *p* = 0.02), and insulin resistance as HOMA-IR (Delta: 1.4 ± 0.1 units; *p* = 0.02). The remaining biochemical parameters were not related to the genotype of obese patients. The percentages of individuals with MS (OR = 2.07, 95% CI = 1.3–3.88; *p* = 0.01), hypertriglyceridaemia (OR = 2.66, 95% CI = 1.43–5.01; *p* = 0.01), and hyperglycaemia (OR = 3.31, 95% CI = 1.26–8.69; *p* = 0.02) were higher in GG subjects than patients with A allele. Logistic regression analysis reported an important risk of the presence of metabolic syndrome in GG subjects (OR = 1.99, 95% CI = 1.21–4.11; *p* = 0.02) after adjusting for adiponectin, dietary energy intakes, gender, weight, and age. **Conclusions**: The GG genotype of rs17300539 is associated with hypertriglyceridaemia, insulin resistance, low adiponectin levels, and a high risk of metabolic syndrome and its components.

## 1. Introduction

Metabolic syndrome is an important group of risk factors with rising prevalence worldwide: glucose intolerance or diabetes mellitus, central obesity, dyslipidaemia (high triglyceride levels and low HDL-cholesterol levels), high blood pressure levels, and an unknown chronic pro-inflammatory state [1]. The metabolic syndrome is the condition that precedes obesity, diabetes mellitus, and all cardiovascular events with high mortality in our Western societies, such as ischemic heart disease, cerebrovascular accident, and peripheral ischemic arteriopathy [2]. In the development of metabolic syndrome are several implied factors presenting a polygenic status and multifactorial disease due to the interaction of an elevated number of different genes (genetic background) with environmental factors (exposome) [2,3]. The genes related to metabolic syndrome involve multiple metabolic pathways related to adipocytokines and sometimes other pathways [3], and, without a doubt, environmental factors such as sedentary lifestyle and excessive caloric intake are factors that influence its development [2]. In this sense, obesity is one of the main factors and is a heterogeneous entity where multiple factors, especially related to physical activity, dietary intake, and genetics as well as other environmental influences contribute to the pathogenicity of obesity. Heritability contributes significantly to obesity development, besides numerous genetic variants associated with various aspects of obesity have been identified in different type of designs [4,5,6].

In this situation, adipose tissue is regarded as an endocrine organ and it has a main role to the development of MS [7]. Adipose tissue has the capacity to generate numerous adipokines, including adiponectin, which has garnered significant interest for its beneficial impact on type 2 diabetes and metabolic disorders since its discovery. It has drawn important attention due to its positive action against the development of type 2 diabetes mellitus and its anti-atherogenic properties and anti-inflammatory effects; these properties allow to prevent cardiovascular events [8,9]. Despite variations in the results of various studies, there is compelling evidence that adiponectin enhances insulin sensitivity by improving lipid and glucose metabolism. Several potential genes have been examined in this area [10,11,12], with single nucleotide polymorphisms accounting for only 5–10% of plasma adiponectin levels thus far. The primary gene associated with levels of circulating adiponectin was *ADIPOQ* on chromosome 3. This gene has emerged as a significant signal in multiple genome-wide association studies conducted among Caucasian populations [13]. On the other hand, associations with metabolic syndrome and/or adiponectin levels have been described for genetic polymorphisms in the *ADIPOQ* gene in some clinical investigations [14,15,16,17,18]. Moreover, some of these relationships could not be reconfirmed by other investigators [19,20,21]. One of the variants is the SNP rs17300539, which has been associated with metabolic response after a weight reduction program in obese patients [22] or fish oil supplementation with pills [23] and with the risk of development diabetes mellitus [24]. As previously mentioned, research conducted by Goyenechea et al. in obese patients [22] revealed that a 30% reduction in energy intake over 8 weeks resulted in improved metabolic parameters in these subjects. Interestingly, their findings indicated that those with the A allele exhibited a protective effect against metabolic risk during dietary intervention over 1 year, as well as protection against repeated weight gain. This fact suggests an intriguing potential interaction between energy intake and the metabolic pathways influenced by rs17300539. Moreover, further insights into this genetic variant’s influence on metabolism were uncovered by Alsaleh et al. [23] who analyzed data from the MARINA study (Modulation of Atherosclerosis Risk by Increasing Doses of n3 Fatty acids). Their work demonstrated an association between rs17300539 and serum adiponectin concentrations, suggesting that the type of dietary fats consumed may also play a role in modulating the SNP’s impact on metabolism pathways of patients with obesity at risk of metabolic syndrome. Despite all of these relationships with metabolic syndrome and dietary interventions with secondary metabolic changes in patients with obesity, the association of this genetic variant rs17300539 with metabolic disorders and adiponectin levels has not been sufficiently evaluated in the literature [24]. For everything previously mentioned, it is very important to have scientific data in this area of knowledge at a time where personalized medicine, and, within it, personalized nutrition, is playing a relevant role in the care of obese patients with a high risk to develop metabolic syndrome and other comorbidities.

Given its potential role in metabolic syndrome, criteria of this entity and serum adiponectin levels, the present investigation was realized to evaluate the role of the SNP rs17300539 in the *ADIPOQ* gene and its relationships with obesity, metabolic syndrome, and serum adiponectin levels in a population of Caucasian out patients with obesity received in an Obesity UNit.

## 2. Materials and Methods

### 2.1. Subjects and Clinical Investigation

Males and females were recruited from a Caucasian population which attended an obesity unit in a fasting state; these subjects were sent to our Nutrition Clinic to treat their weight problem (body mass index ≥ 30 kg/m^2^). A total of 329 subjects with obesity agreed to be enrolled in the trial and all patients gave their signed informed consent for enrollment before they were included in the present trial. The investigation was conducted in accordance with the Declaration of Helsinki and the Local Ethics Committee of our hospital (No 01/2022) approved the study. The inclusion criteria of our study were as follows: body mass index ≥ 30 kg/m^2^, no previous history of cardiovascular events, no active alcoholism, no malignant tumors, did not receive any medications known to influence lipid or glucose levels within the 12 weeks before the study, and followed a hypocaloric diet during the pre-recruitment period (less than 6 months). Patients taking drugs for hyperlipidemia, hypertension, obesity, or diabetes mellitus were excluded.

During the enrollment visit and after signing the informed consent, the next adiposity parameters (weight, body mass index (BMI), fat mass by bio impedance and waist circumference), systolic and diastolic blood pressure, and dietary intake were recorded. In the same basal visit, a total of 10 mL of venous blood was aliquoted in ethylenediaminetetraacetic acid (EDTA)-coated tubes in a fasting state of 10 h, with the patient lying on a medical stretcher and after 10 min of rest. The next biochemical parameters were also determined: insulin, lipid profile as (serum total cholesterol, serum LDL-cholesterol, serum HDL-cholesterol and serum triglycerides), serum adiponectin, and C-reactive protein. Finally, all patients were genotyped (rs17300539). To genotype patients, a peripheral blood sample, a total of 5 mL, is used to extract DNA from circulating lymphocytes.

MS was identified based on the criteria set by the Adult Treatment Panel III. Patients had to comply at least three of the following criteria to receive an metabolis syndrome diagnosis: high fasting glucose or diabetes treatment, elevated triglycerides (above 150 mg/dL) or dyslipidemia treatment, low HDL cholesterol below 40 mg/dL (males) or below 50 mg/dL (females), high systolic or diastolic blood pressure (above 130/85 mmHg), and increased waist circumference [1].

### 2.2. Adiposity Parameters and Blood Pressure

Height (cm) and waist diameter (cm) were determined using a non-elastic measuring tape (Omrom, Los Angeles, CA, USA). For all patients, their unclothed body weight was determined using digital scales (Omrom, Los Angeles, CA, USA). Body mass index (BMI) was calculated according to the measurement of total body weight (kg) divided by the square of height (m). Total body fat mass was determined by bio impedance [25] (EFG BIA 101 Anniversary, Akern, Pisa, Italy) using the next equation: (0.756 Height^2^/Resistance) + (0.110 Body mass) + (0.107 Reactance) − 5.463.

Diastolic and systolic blood pressures were assessed through the average of three consecutive determinations taken (Omrom, Los Angeles, CA, USA) after the participants had been seated for 10 min.

### 2.3. Biochemical Procedures

The lipid levels, glucose levels, and inflammatory markers such as C-reactive protein and adiponectin were analyzed in the blood sample. The COBAS INTEGRA 400 analyzer was used to measure total cholesterol, HDL cholesterol, and triglyceride levels. LDL cholesterol was calculated indirectly by the Friedewald equation (LDL cholesterol = total cholesterol − HDL cholesterol − triglycerides/5) [26].

Fasting glucose levels were measured by a total automated hexoquinase oxidase method and basal insulin was determined using the electrochemiluminescence assay (COBAS INTEGRA 400 analyzer, Roche Diagnostic, Montreal, QC, Canada). Subsequently, the homeostasis model assessment for insulin resistance (HOMA-IR) was obtained using both parameters with the following equation (glucose × insulin/22.5) [27]. C-reactive protein (CRP) was measured by a standard method of immunoturbimetry (Roche Diagnostics GmbH, Mannheim, Germany).

Finally, serum adiponectin levels were determined by Enzyme Linked ImmunoSorbent Assay (ELISA) with a commercial kit (R&D systems, Inc., Mineapolis, MN, USA) (DRP300).

### 2.4. Genotyping of the ADIPOQ Gene

DNA was isolated from oral swabs using QIAamp^®^ (Hilden, Germay). The −11391 G/A rs17300539 SNP was analyzed in all participants utilizing a technique based on quantitative DNA polymerase chain reaction (qPCR) (QuantStudio 12K Flex Real-Time qPCR instrument, Thermofisher, Pittsburgh, PA, USA). Evaluation of genotyping accuracy involved the incorporation of duplicates in the arrays and inclusion of negative controls (water) on each plate. In the real time polymerase chain reaction, we used a final volume of 10 μL containing 2.5 μL TaqMan OpenArray Master Mix (Applied Biosystems, Foster City, Los Angeles, CA, USA) and 2.5 μL human DNA sample according to the manufacturer’s instructions (Thermocycler Life Technologies, Los Angeles, CA, USA). Genotype calling and sample clustering for Open Array assays was performed using the TaqMan Genotyper (Life Technologies, Carlsbad, CA, USA). The Hardy–Weinberg equilibrium was assessed using a statistical test. The *ADIPOQ* gene variant was found to be in Hardy–Weinberg equilibrium (*p* = 0.47).

### 2.5. Dietary Intakes and Physical Activity

For all patients, a dietitian recorded the dietary intakes on three non-consecutive days in order to evaluate the total daily intake of calories and macronutrient distribution. In the evaluation of the dietary intake of these three days, two days were included, that is, between Monday and Friday and a weekend day (Saturday or Sunday); in this way, the average obtained intakes will better reflect the real intake of seven days a week. The dietary registrations were determined using professional software (Dietsource^®^, version 1.0, Nestlé, Geneve, Switzerland) [28]. Physical activity was self-reported by each subject using a questionnaire. In this questionnaire, the patient noted their daily physical activity in minutes and an average of that activity was recorded for the 7 days of the week, with a final result in minutes/week.

### 2.6. Statistical Analysis

The study’s sample size was established to detect total body weight differences of over 3 kg with 90% power and 5% significance (n = 320). The distribution of variables was assessed using the Shapiro–Wilk test and Kolmogorov–Smirnov test. The *ADIPOQ* rs17300539 genotype was analyzed using a dominant model (GG vs. GA + AA), in this model, the assumption is that the presence of a single A allele is already a risk allele. Analysis of the Student t-test and Kruskal–Wallis test was fitted for parametric and nonparametric data, respectively. The Bonferroni test was applied for multiple testing to reduce Type I errors in association analysis.

Logistic regression analyses were adjusted for adiponectin, energy intake, age, sex, and weight to calculate odds ratios (ORs) and the 95% confidence interval (CI) for estimating the association of the SNP with components of metabolic syndrome. The statistical analysis was conducted using SPSS version 23.0 for Windows software package. Statistical significance was defined as *p* values below 0.05. (SPSS Inc., Chicago, IL, USA).

## 3. Results

A group of 329 Caucasian subjects with obesity formed the total study sample. Individuals were middle aged with a mean age of 47.8 ± 2.1 years (range: 29–55) and a mean body mass index (BMI) of 39.8 ± 2.9 kg/m^2^ (range: 36.7–41.1), respectively. Gender distribution was 232 females (70.5%) and 97 males (29.5%), with a clear predominance of women in the selected sample. The percentage of different genotypes of the rs17300539 polymorphism in this sample was 86.0% (n = 283) (GG), 11.2% (n = 37) (GA), and 2.7% (n = 9) (AA). The evaluation of these genotypes showed the next allele frequency; G allele (0.76) and A allele (0.24) (risk allele).

The average age and mean BMI were equal in both of the above-mentioned genotype groups. Mean values of age (GG; 48.0 ± 1.1 years vs. GA + AA; 47.6 ± 2.0 years: ns) as well as the female/male ratio (GG 28% males vs. 72% females vs. GA + AA; 33% males vs. 67% females: ns) were similar between both genotype groups.

According to our dominant genetic model evaluation, Table 1 shows the average values for anthropometric parameters and blood pressure data (mean ± SD). When analyzing the two genotypes, all anthropometric parameters were similar without statistical differences between groups. Systolic and diastolic blood pressures remained similar in both genotype groups.

Biochemical parameters and serum adiponectin levels related to genotype are shown in Table 2. Analyzing both genotypes, the next values (triglycerides, insulin, and HOMA-IR) were lower in AA + GA patients than GG patients (Table 2). Applying this dominant genetic model again (GG vs. GA *+* AA), we noted statistical disparities between the genotype groups in their serum adiponectin levels (Delta: 7.5 ± 1.4 ng/mL; *p* = 0.03), triglycerides (Delta: 41.1 ± 3.4 mg/dL; *p* = 0.01), insulin (Delta: 4.9 ± 1.1 mUI/L; *p* = 0.02), and HOMA-IR (Delta: 1.4 ± 0.1 units; *p* = 0.02).

Table 3 presents the daily dietary intakes and self-reported physical activity. According to the dominant model, there were no statistically significant differences in average carbohydrate, lipid, and protein intakes. The profile of the type of dietary fat (saturated, monounsaturated, and polyunsaturated) are likewise similar. And finally, fiber intakes between genotype GG vs. genotype GA + AA (dominant model) were equal. Moreover, energy intake was higher in the GG group (Delta: 285.1 ± 5.4 cal/day; *p* = 0.02). Physical activity was also similar in genotype GG vs. genotype GA + AA (dominant model).

According to the metabolic characteristics results, 62% of individuals (n = 204) had metabolic syndrome, while 38% of patients (n = 125) did not have this entity. The frequency of subjects with metabolic syndrome and various components of this entity (central obesity, hypertriglyceridemia, hypertension, or hyperglycemia) is presented in Table 4. Percentage of patients with hypertriglyceridemia, patients with hyperglycemia were higher in subjects with A allele with statistical differences. According to the results of metabolic characteristics and calculating odss ratios, the percentages of individuals who had metabolic syndrome (OR = 2.07, 95% CI = 1.3–3.88; *p* = 0.01), hypertriglyceridemia (OR = 2.66, 95% CI = 1.43–5.01; *p* = 0.01), and hyperglycemia (OR = 3.31, 95% CI = 1.26–8.69; *p* = 0.02) were higher in GG subjects than in individuals with the A allele.

The cutoff points for defining central obesity (waist circumference > 88 cm in females and >102 in males), hypertension (systolic BP > 130 mmHg or diastolic BP > 85 mmHg or specific treatment), hypertriglyceridemia (triglycerides > 150 mg/dL or specific treatment), or hyperglycemia with fasting plasma glucose level of >110 mg/dL, taking drugs to treat elevated blood sugar. * *p* < 0.05 among both genotypes compared using a dominant model (GG vs. GA + AA).

Logistic regression statistical analysis showed an increased risk of percentage of metabolic syndrome in GG subjects (OR = 1.99, 95% CI = 1.21–4.11; *p* = 0.02) after adjusting for serum adiponectin, dietary energy intakes, gender, weight, and age of the patients with obesity.

## 4. Discussion

In our present study, the action of the adiponectin gene (*ADIPOQ*) promoter SNP −11391 G/A (rs17300539) has been demonstrated on the risk of metabolic syndrome presence, insulin resistance, hypertriglyceridemia, and low serum adiponectin levels in Caucasian patients with obesity.

First, we observed that the frequency of the minor allele (A) was equal to that reported in other general Caucasian populations in previous studies [17,29]. For this reason, we consider our study to be representative, which allows us to carry out an evaluation of the association with the different parameters analysed in our design. In the literature, the GG genotype has been linked to an increased susceptibility to type 2 diabetes mellitus and insulin resistance [16,30]. However, there is a shortage of studies that have examined its association with metabolic syndrome, the entity which precedes type 2 diabetes mellitus. Identifying high-risk patients is therefore a focus area for preventing the development of type 2 diabetes mellitus.

From a pathophysiological point of view, insulin resistance is considered the main defect of metabolic syndrome and *ADIPOQ* gene variants could be involved in this susceptibility. This potential association is due to a reduced serum adiponectin levels and increased fasting circulating insulin, which is attributed to the role of adiponectin in decreasing triglyceride content in different tissues and enhancing insulin signaling [31]. In our design, the GG genotype was related to an increased risk of MS and specifically with two criteria of this entity: hyperglycemia and hypertriglyceridemia. The main hypothesis of this association is that the *ADIPOQ* variant affects insulin resistance by regulating serum adiponectin levels [32]. Some studies have reported decreased levels related to the G allele of the rs17300539 variant [29,30], while other investigations have detected no association between the genetic variant and serum adiponectin levels [21]. It is important to consider different sources of information when evaluating these findings. Some research has indicated reduced levels connected to the G allele of the rs17300539 variation, while others have found no link between the genetic variant and adiponectin levels [21,29,30]. Its position was five inches from the response element for the transcriptional activator PPAR gamma (peroxisome proliferator response element) in the *ADIPOQ* promoter region implies a potential impact on transcription amount. In previous studies, Bouatia et al. [17] reported that the G allele had lower *ADIPOQ* promoter activity in COS7 cells, compatible with the lower circulating adiponectin levels.

A strength of the present work is the evaluation of dietary intake, showing a decrease in total energy intake in patients with the A allele. This dietary association has been described previously in the literature, specifically for the rs17300539 variant [33]. A prospective study among non-Caucasian children found that children with the A allele had a lower energy intake per day than those with the GG genotype [34]. However, in this work, the different macronutrients in the diet were not evaluated and the intake of different dietary lipids (saturated, monounsaturated, and polyunsaturated) was also not recorded, preventing definitive conclusions to be drawn about this association with the energy intake found. In our work, we also found an increase in caloric intake in patients with obesity and the GG genotype, with a non-significant increase in specific macronutrients in general. In the literature, there are also nutritional intervention studies in which we can also see some relationship with intake and this genetic variant. For example, Goyenechea et al. [22] demonstrated that a 30% energy restriction for 8 weeks improved metabolic parameters in overweight patients, but those with the A allele have a protective action to recover the metabolic risk after dietary intervention at 32 and 60 weeks; also, this allele confers protection to a new weight gain. Therefore, these results indicate a potential interaction between energy intake and the metabolic pathways in which rs17300539 acts. The type of dietary fat ingested may also influence the association of this SNP with metabolic parameters, as demonstrated by Alsaleh et al. [23] when analyzing the results of the MARINA study (Modulation of atherosclerosis Risk by Increasing Doses of n3 Fatty acids), for example. In this study [23], an interaction between rs17300539 and serum adiponectin concentrations with dietary n3 polyunsaturated fatty acids was reported. Unsaturated fatty acids act as activators for the transcription factor PPAR gamma [17], leading to an increase in *ADIPOQ* gene expression and a subsequent increase in serum levels of circulating adiponectin.

Finally, other studies have also shown an elevation of total cholesterol levels in patients with extreme obesity and the presence of GG genotype [35]. In our study, this potential association was not found. In the study by Gasparotto et al. [35], the sample of patients had an average BMI of 50 kg/m^2^, which may explain the differences with our study. This sample of the obese population increases by more than 10 points in BMI compared to our studied sample. The contradictory results in the above-mentioned investigations from the literature may be due to the type of population analysed: different ethnicities, the presence or absence of diabetes mellitus in the patients, different degrees of obesity, and different types of basal diets and amount of physical exercise [36].

Our study has some limitations that should be taken into consideration. Firstly, it was conducted on Caucasian patients with moderate obesity, thus the findings may not be applicable to all populations and all ethnicities. For example, in a population of overweight females with polycystic ovary syndrome, a lack of association between this *ADIPOQ* genetic variant and metabolic syndrome and comorbidities was recently reported [37]. Secondly, the study design was performed as a cross-sectional study, so we cannot provide causality in the same way as previous interventional studies or longitudinal follow-up cohort studies. Finally, dietary intake and daily physical activity have been carried out with surveys reported by the patients themselves, which can produce certain estimation biases.

## 5. Conclusions

In conclusion, this investigation was able to confirm that the GG genotype of *ADIPOQ* rs17300539 is associated with higher insulin resistance, triglyceride levels, and percentage of patients with metabolic syndrome compared with patients carried the A allele. Patients with this genotype had lower circulating adiponectin, which could be implied in the above-mentioned metabolic risk factors. Further designs are needed to reconfirm this relationship, especially because patients with obesity and lack of the A allele have a high cardiovascular risk and early preventive actions would be required to avoid cardiovascular events and the development of diabetes mellitus. In the era of personalized medicine and therefore personalized nutrition, the ability to predict metabolic risk and responses to therapies is very important, hence the relevance of our scientific findings. This personalization of medicine and nutrition is very important in a pathology like obesity that is reaching pandemic proportions around the world.

## Figures and Tables

**Table 1:** Anthropometric-adiposity parameters and blood pressure determinations.

Parameters	GG n = 283	GA + AA n = 46	*p*
BMI	40.9 ± 2.1	39.8 ± 1.9	0.23
Weight (kg)	106.8 ± 2.0	105.9 ± 2.8	0.32
Fat mass (kg)	49.9 ± 1.9	48.9 ± 2.1	0.42
WC (cm)	122.1 ± 3.0	121.8 ± 2.3	0.23
SBP (mmHg)	128.7 ± 1.9	127.3 ± 2.1	0.39
DBP (mmHg)	80.1 ± 4.0	79.2 ± 4.1	0.38

BMI: body mass index DBP, diastolic blood pressure; SBP, systolic blood pressure; WC, waist circumference. No significant variances. A dominant model (GG vs. GA + AA).

**Table 2:** **Basal** Biochemical parameters expressed as mean ± SD.

Parameters	GG n = 283	GA + AA n = 46	*p*
Fasting Glucose (mg/dL)	101.2 ± 2.0	102.1 ± 3.0	0.34
Total cholesterol (mg/dL)	195.3 ± 10.8	195.2 ± 9.0	0.21
LDL-cholesterol (mg/dL)	114.3 ± 3.1	114.4 ± 4.0	0.38
HDL-cholesterol (mg/dL)	52.9 ± 2.1	53.1 ± 2.0	0.32
Triglycerides (mg/dL)	153.1 ± 10.9	112.7 ± 8.4 *	0.01
Insulin (mUI/L)	21.0 ± 2.1	16.1 ± 1.9 *	0.02
HOMA-IR	5.5 ± 0.4	4.1 ± 0.1 *	0.02
CRP (mg/dL)	4.7 ± 0.4	4.5 ± 0.8	0.41
Adiponectin (ng/mL)	9.7 ± 0.4	17.2 ± 0.9	0.03

HOMA-IR (homeostasis model assessment of insulin resistance). CRP C reactive protein. * *p* < 0.05 among both genotypes in a dominant model (GG vs. GA + AA).

**Table 3:** Dietary intakes distribution and physical activity (mean ± SD).

Parameters	GG n = 283	GA + AA n = 46	*p*
Calories (cal/day)	1887.3 ± 100.2	1601.5 ± 90.5 *	0.02
Carbohydrates (g/day)	215.3 ± 12.1	200.2 ± 15.3	0.15
Proteins (g/day)	89.9 ± 9.1	73.6 ± 2.4	0.21
Lipids (g/day)	74.6 ± 4.1	65.2 ± 3.1	0.31
Fiber (g/day)	12.4 ± 3.0	14.9 ± 1.1	0.29
Cholesterol (mg/day)	207.6 ± 12.9	208.7 ± 19.8	0.21
Saturated fatty acids (g/day)	37.5 ± 3.2	36.8 ± 4.0	0.49
Monounsaturated fatty acids (g/day)	30.1 ± 4.2	27.2 ± 3.1	0.41
Polyunsaturated fatty acids (g/day)	8.1 ± 4.0	7.0 ± 3.8	0.28
Physical activity (minutes/week)	103.9 ± 12.7	101.9 ± 10.1	0.33

* *p* < 0.05 among both genotypes in a dominant model (GG vs. GA + AA).

**Table 4:** Metabolic syndrome (MS) and components of MS.

Parameters	GG n = 283	GA + AA n = 46	*p*
Percentage of MS	62.0%	47.8% *	0.01
Percentage of central obesity	65.4%	52.1%	0.24
Percentage of hypertriglyceridaemia	69.1%	45.6% *	0.01
Low HDL cholesterol	23.1%	17.9%	0.23
Percentage of hypertension	75.5%	76.1%	0.61
Percentage of hyperglycaemia	28.8%	10.8% *	0.02

## Data Availability

All data generated or analysed during this study are included in this article. Further enquiries can be directed to the corresponding author.

## References

[1] Expert Panel on Detection, Evaluation and Treatment of High Blood Cholesterol in Adults (Adult Treatment Panel III) (2001). Executive summary of the Third Report of the National Cholesterol Education Program (NCEP). JAMA.

[2] Portela-Cidade J.P., Borges-Canha M., Leite-Moreira A.F., Pimentel-Nunes P. (2015). Systematic review of the relation between intestinal microbiota and toll-like receptors in the metabolic syndrome: What do we know so far?. GE Port. J. Gastroenterol..

[3] de Luis D.A., Izaola O., Primo D., Aller R. (2020). Relation of a variant in adiponectin gene (rs266729) with metabolic syndrome and diabetes mellitus type 2 in adult obese subjects. Eur. Rev. Med. Pharmacol. Sci..

[4] Elks C.E., den Hoed M., Zhao J.H., Sharp S.J., Wareham N.J., Loos R.J.F. (2012). Variability in the heritability of body mass index: A systematic review and meta-regression. Front. Endocrinol..

[5] de Luis D.A., Izaola O., Primo D. (2021). The lactase rs4988235 is associated with obesity related variables and diabetes mellitus in menopausal obese females. Eur. Rev. Med. Pharmacol. Sci..

[6] de Luis D.A., Izaola O., Primo D., de la Fuente B., Aller R. (2017). Polymorphism rs3123554 in the cannabinoid receptor gene type 2 (CNR2) reveals effects on body weight and insulin resistance in obese subjects. Endocrinol. Diabetes Nutr..

[7] Sheng T., Yang K. (2008). Adiponectin and its association with insulin resistance and type 2 diabetes. J. Genet. Genom..

[8] Matsuzawa Y., Funahashi T., Kihara S., Shimomura I. (2004). Adiponectin and metabolic syndrome. Arterioscler. Thromb. Vasc. Biol..

[9] Matsuzawa Y. (2006). The metabolic syndrome and adipocytokines. FEBS Lett..

[10] Breitfeld J., Stumvoll M., Kovacs P. (2012). Genetics of adiponectin. Biochimie.

[11] Hu E., Liang P., Spiegelman B.M. (1996). AdipoQ is a novel adipose-specific gene dysregulated in obesity. J. Biol. Chem..

[12] Crimmins N.A., Martin L.J. (2007). Polymorphisms in adiponectin receptor genes ADIPOR1 and ADIPOR2 and insulin resistance. Obes. Rev..

[13] Jee S.H., Sull J.W., Lee J.-E., Shin C., Park J., Kimm H., Cho E.-Y., Shin E.-S., Yun J.E., Park J.W. (2010). Adiponectin concentrations: A genome-wide association study. Am. J. Hum. Genet..

[14] Hara K., Boutin P., Mori Y. (2002). Genetic variation in the gene encoding adiponectin is associated with an increased risk of type 2 diabetes in the Japanese population. Diabetes.

[15] Menzaghi C., Ercolino T., Di Paola R. (2002). A haplotype at the adiponectin locus is associated with obesity and other features of the insulin resistance syndrome. Diabetes.

[16] Vasseur F., Helbecque N., Dina C., Lobbens S., Delannoy V., Gaget S., Boutin P., Vaxillaire M., Lepretre F., Dupont S. (2002). Single-nucleotide polymorphism haplotypes in the both proximal promoter and exon 3 of the APM1 gene modulate adipocyte-secreted adiponectin hormone levels and contribute to the genetic risk for type 2 diabetes in French Caucasians. Hum. Mol. Genet..

[17] Bouatia-Naji N., Meyre D., Lobbens S., Seron K., Fumeron F., Balkau B., Heude B., Jouret B., Scherer P.E., Dina C. (2006). ACDC/adiponectin polymorphisms are associated with severe childhood and adult obesity. Diabetes.

[18] Heid I.M., Wagner S.A., Gohlke H. (2006). Genetic architecture of the APM1 gene and its influence on adiponectin plasma concentrations and parameters of the metabolic syndrome in 1,727 healthy Caucasians. Diabetes.

[19] Filippi E., Sentinelli F., Trischitta V. (2004). Association of the human adiponectin gene and insulin resistance. Eur. J. Hum. Genet..

[20] Ohashi K., Ouchi N., Kihara S. (2004). Adiponectin I164T mutation is associated with the metabolic syndrome and coronary artery disease. J. Am. Coll. Cardiol..

[21] de Courten B.V., Hanson R.L., Funahashi T., Lindsay R.S., Matsuzawa Y., Tanaka S., Thameem F., Gruber J.D., Froguel P., Wolford J.K. (2005). Common polymorphisms in the adiponectin gene ACDC are not associated with diabetes in Pima Indians. Diabetes.

[22] Goyenechea E., Collins L.J., Parra D., Abete I., Crujeiras A.B., O’Dell S.D., Martínez J.A. (2009). The -11391 G/A polymorphism of the adiponectin gene promoter is associated with metabolic syndrome traits and the outcome of an energy-restricted diet in obese subjects. Horm. Metab. Res..

[23] Alsaleh A., Crepostnaia D., Maniou Z., Lewis F.J., Hall W.L., Sanders T.A., O’Dell S.D. (2013). MARINA study team. Adiponectin gene variant interacts with fish oil supplementation to influence serum adiponectin in older individuals. J. Nutr..

[24] Howlader M., Sultana M.I., Akter F., Hossain M.M. (2021). Adiponectin gene polymorphisms associated with diabetes mellitus: A descriptive review. Heliyon.

[25] Lukaski H., Johson P.E. (1985). Assessment of fat-free mass using bioelectrical impedance measurements of the human body. Am. J. Clin. Nutr..

[26] Friedewald W.T., Levy R.J., Fredrickson D.S. (1972). Estimation of the concentration of low-density lipoprotein cholesterol in plasma without use of the preparative ultracentrifuge. Clin. Chem..

[27] Mathews D.R., Hosker J.P., Rudenski A.S., Naylor B.A., Treacher D.F. (1985). Homeostasis model assessment: Insulin resistance and beta cell function from fasting plasma glucose and insulin concentrations in man. Diabetologia.

[28] Mataix J., Mañas M. (2003). Tablas de Composición de Alimentos Españoles.

[29] Petrone A., Zavarella S., Caiazzo A., Leto G., Spoletini M., Potenziani S., Osborn J., Vania A., Buzzetti R. (2006). The promoter region of the adiponectin gene is a determinant in modulating insulin sensitivity in childhood obesity. Obesity.

[30] Schwarz P.E., Towers G.W., Fischer S., Govindarajalu S., Schulze J., Bornstein S.R., Hanefeld M., Vasseur F. (2006). Hypoadiponectinemia is associated with progression toward type 2 diabetes and genetic variation in the ADIPOQ gene promoter. Diabetes Care.

[31] Kissebah A.H., Sonnenberg G.E., Myklebust J., Goldstein M., Broman K., James R.G., Marks J.A., Krakower G.R., Jacob H.J., Weber J. (2000). Quantitative trait loci on chromosomes 3 and 17 influence phenotypes of the metabolic syndrome. Proc. Natl. Acad. Sci. USA.

[32] Heliovaara M.K., Strandberg T.E., Karonen S.L., Ebeling P. (2006). Association of serum adiponectin concentration to lipid and glucose metabolism in healthy humans. Horm. Metab. Res..

[33] Kroll C., Mastroeni S.S.B.S., Veugelers P.J., Mastroeni M.F. (2019). Associations of *ADIPOQ* and *LEP* gene variants with energy intake: A systematic review. Nutrients.

[34] Zandona M.R., Rodrigues R.O., Albiero G., Campagnolo P.D., Vitolo M.R., Almeida S., Mattevi V.S. (2013). Polymorphisms in LEPR, PPARG and APM1 genes: Associations with energy intake and metabolic traits in young children. Arq. Bras. Endocrinol. Metabol..

[35] Gasparotto A.S., Borges D.O., Zandoná M.R., Ramos M.J., Meihnardt N.G., Mattevi V.S. (2017). Adiponectin promoter polymorphisms are predictors of lipid profile improvement after bariatric surgery. Genet. Mol. Biol..

[36] Coltell O., Ortega-Azorín C., Sorlí J.V., Portolés O., Asensio E.M., Saiz C., Barragán R., Estruch R., Corella D. (2021). Circulating adiponectin and its association with metabolic traits and type 2 diabetes: Gene-diet interactions focusing on selected gene variants and at the genome-wide level in high-cardiovascular risk Mediterranean subjects. Nutrients.

[37] Nowak I., Ciećwież S., Łój B., Brodowski J., Brodowska A. (2021). Adiponectin gene polymorphism (rs17300539) has no influence on the occurrence of metabolic syndrome in women with polycystic ovary syndrome. Genes.

